# Non-interferometric photoacoustic remote sensing microscopy

**DOI:** 10.1038/lsa.2016.278

**Published:** 2017-06-02

**Authors:** Parsin Hajireza, Wei Shi, Kevan Bell, Robert J Paproski, Roger J Zemp

**Affiliations:** 1 Department of Electrical and Computer Engineering, University of Alberta, Edmonton, Alberta, T6G 2V4 Canada; 2 IllumiSonics, Inc., 5205-38A Ave. N.W., Edmonton, Alberta, T6L 2J4, Canada

**Keywords:** all optical, medical imaging, optical resolution, photoacoustic, remote sensing

## Abstract

Elasto-optical refractive index modulation due to photoacoustic initial pressure transients produced significant reflection of a probe beam when the absorbing interface had an appreciable refractive index difference. This effect was harnessed in a new form of non-contact optical resolution photoacoustic microscopy called photoacoustic remote sensing microscopy. A non-interferometric system architecture with a low-coherence probe beam precludes detection of surface oscillations and other phase-modulation phenomenon. The probe beam was confocal with a scanned excitation beam to ensure detection of initial pressure-induced intensity reflections at the subsurface origin where pressures are largest. Phantom studies confirmed signal dependence on optical absorption, index contrast and excitation fluence. *In vivo* imaging of superficial microvasculature and melanoma tumors was demonstrated with ~2.7±0.5 μm lateral resolution.

## Introduction

Optical imaging of biological specimens has provided biologists and clinicians with valuable tools for science and medicine. Many such techniques rely on fluorescence or optical scattering as contrast mechanisms. Optical absorption is a desirable contrast mechanism because it can provide information about chemical bonds, molecular structure, blood oxygenation, and other biochemical data. Although transmission-mode sensing of optical absorption is possible with thin transparent samples using ballistic imaging techniques or photothermal microscopy^1, 2, 3^, reflection mode imaging of optical absorption requires other methods. Diffuse optical tomography^4^ is capable of estimating subsurface absorption and scattering distributions. However, the spatial resolution is poor, and the associated inverse problems are ill posed and ill conditioned.

Photoacoustic (PA) methods have demonstrated great success in imaging acoustic pressure distributions due to light absorption-induced thermoelastic expansion. These acoustic signals are detected and reconstructed to form images with intrinsic optical absorption contrast. PA imaging provides exquisite images of microvessels^5, 6, 7, 8, 9^, and it is capable of imaging blood oxygen saturation^10, 11, 12^, gene expression^13^ and contrast agents^14, 15^. Both acoustic resolution and optical resolution embodiments have been significant^16, 17, 18^. In most PA and ultrasound imaging systems, piezoelectric transducers have been employed in which an ultrasound coupling medium, such as water or ultrasound gel, is required. However, for many clinical applications, such as wound healing^19^, burn diagnostics^20^, surgery^21^ and many endoscopic procedures^22, 23^, physical contact, coupling, or immersion is undesirable or impractical. Optical methods to detect ultrasound and photoacoustic signals have been investigated over a number of years^24, 25, 26, 27, 28, 29, 30, 31, 32, 33, 34, 35, 36, 37, 38, 39, 40^. Most previous approaches detected surface oscillations with interferometric methods. Others used interferometry to observe photoacoustic stresses, including optical coherence tomography (OCT) methods^27, 34^. These methods offer potential sensitivity to the scattered probe beam phase modulations associated with motion of scatterers, subsurface and surface oscillations, as well as unwanted vibrations. They are also sensitive to complex amplitude reflectivity modulations. The net interferometric signal may be a mixture of these composite mechanisms and could lead to unwanted interference. The proposed approach in the present paper is distinct because we intentionally eliminated phase-sensitivity to exclusively monitor intensity reflectivity changes. In addition, the proposed system has the potential to be real-time, unlike reported OCT photoacoustic imaging systems, which require mechanical depth scanning, leading to slower acquisition rates^34^. This work introduces a new mechanism and methodology to detect photoacoustic signals at the subsurface origin, where pressures are maximal. Elasto-optical refractive index changes due to photoacoustic initial pressure transients are shown to produce a significant time-varying reflection of a probe beam when the absorbing interface also has an appreciable refractive index contrast. Intensity-reflection coefficient modulations are negligible without such a static refractive index difference.

To observe such reflection modulations, the intensity changes of a probe beam in response to a generated photoacoustic initial pressure are measured. A non-interferometric approach with a low-coherence probe beam precludes any phase-modulation sensitivity to enable detection of intensity variations. The proposed approach transiently amplifies existing refractive index steps where absorption is present. While effective static signals from the probe beam occur due to inherent scattering and reflection, modulations are present only when photoacoustic initial pressures are generated. The non-interferometric photoacoustic remote sensing (PARS) microscope presented here takes advantage of sensitive subsurface initial pressure detection to achieve very high signal-to-noise ratios with a ~2.5 cm working distance from the objective lens to the sample, enabling high-quality, real-time *in vivo* imaging.

## Materials and methods

The refractive index inside a medium can be modulated due to significant changes in pressure or temperature. In photoacoustics, the expected magnitude of local initial pressure is large (>MPa) and can be calculated assuming optical focusing and thermal confinement conditions are applied. Initial pressure is given as 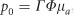, where 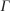 is the Grüneisen parameter, *μ*_*a*_ is the optical absorption coefficient and *Φ* is the incident fluence^1^. The generated step-like pressure increase modulates the existing refractive index *n*_0_(*x*) of the medium by an amount *δn*(*x*,*t*) following the elasto-optic relation^41^:


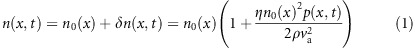


where η is the elasto-optic coefficient (~0.32 for water), *p*(*x*,*t*) is the pressure field, *ρ* is mass density and *v*_a_ is the speed of sound in the medium.

**Figure 1:**
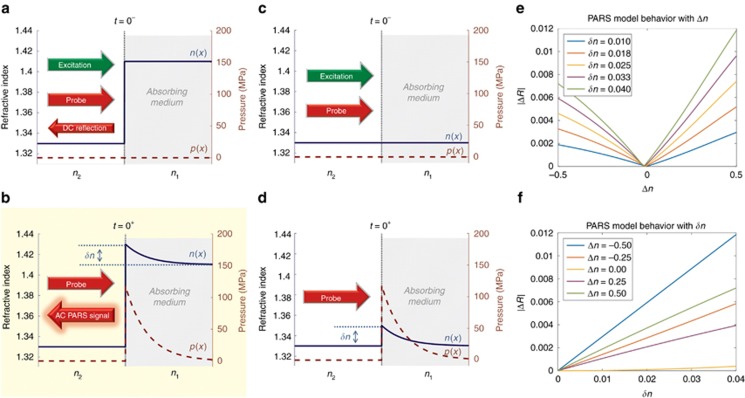
Diagram of the PARS mechanism. (**a**) If a refractive index profile *n*(*x*) exists at the boundary of an absorbing media having a refractive index *n*_1_, a DC reflection of a probe beam is expected before pulsed excitation occurs (*t*=0^-^). (**b**) The excitation beam has been absorbed (*t*=0^+^), and thermoelastic excitation of the absorber generates an initial pressure profile *p*(*x*). This, in turn, perturbs the existing refractive index by *δn*, which produces an AC modulation in the reflected probe beam proportional to the absorption. Note that the DC component is not shown in this figure for simplicity. (**c**, **d**) If there is no existing refractive index step, then the small *δn* modulation generated by absorption of the excitation beam creates a negligible AC signal variation (not shown). (**e**) The behavior of Equation 2 versus the static refractive index step, Δ*n*=*n*_1_−*n*_2_. (**f**), A sweep of Equation 2 versus the modulation *δn*. The linearity of Δ*R* with *δn* and Δ*n* is clearly demonstrated.

For whole blood, the absorption coefficient is approximately 237 cm^−1^ at 532 nm. With a Grüneisen parameter close to unity (although some tissues have a Grüneisen parameter closer to 0.2^1^), and with a focal fluence of 500 mJ cm^−^^2^, an initial pressure of approximately 118 MPa is predicted. This results in a modulation of *δn*≈0.020 in the absorbing medium *n*_1_ (Figure 1a–1d). Focal fluence levels simulated here are comparable to those used in optical resolution photoacoustic microscopy (OR-PAM)^42^, and surface fluences can be maintained below maximum permissible exposure limits. Laser safety details are given in Section 1 of the Supplementary Information.

Thermal effects may also change refractive indices. Near 20 °C, the refractive index of water would change ~0.006% per °C. Even assuming a 28 °C temperature rise (*δn*≈0.002), the effect is negligible compared to pressure-induced effects. In addition, thermal cooling will occur on the microsecond to millisecond scale after laser-induced heating, which is significantly slower than the PARS signals observed.

The index modulation alone produces a small change in reflectivity. If the time-varying refractive index is near an existing refractive index step (such that there is negligible loss of pressure in the acoustic wave), a strong modulation of reflectivity can occur at the interface. Assuming that an absorbing medium with refractive index *n*_1_ is being modulated by initial pressure *p*(*x*), it yields a refractive index perturbation δ*n* at an interface with a non-absorbing medium having constant refractive index *n*_2_. The intensity-reflection coefficient perturbation at the boundary and immediately after a short laser pulse is then given as


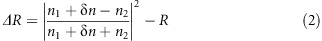


where 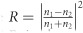 is the unperturbed intensity-reflection coefficient and Δ*R* is the perturbation due to photoacoustic initial pressure. Assuming that δ*n* is primarily real, then the reflectivity perturbation due to photoacoustic initial pressure is given as


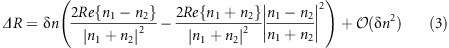


where 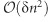 represents the terms that depend on higher orders of *δn*. When refractive indices are primarily real, we obtain the following relationship:


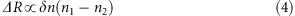


as shown in Figure 1e and 1f. Thus, with a refractive index contrast at the absorption boundary, the intensity reflectivity coefficient is modulated in proportion to the refractive index step 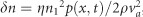, which is in turn proportional to the initial pressure. When *n*_1_≈*n*_2_ (index-matched boundaries) the term inside the brackets on the right-hand side of Equation 3 approaches zero, and the signal is dependent on higher order terms of the small index change *δn*. For the same *δn*=0.020, a reflectivity perturbation of Δ*R*≈5.3 × 10^−5^ is expected when *n*_1_≈*n*_2_ which is an order of magnitude less than when |*n*_1_−*n*_2_|=0.1 (in which case Δ*R*≈6.1 × 10^−4^). Soon after the initial pressure is generated, a finite amplitude step wave will propagate away from the interface, and a more complicated spatio-temporal refractive index profile will be observed at and away from the boundary, resulting in a rapidly attenuated reflectivity modulation.

The system is sensitive to intensity reflectivity modulations at any depth within the probe beam optical depth-of-focus. Such modulations effectively begin instantaneously, coincident with the excitation pulse, irrespective of depth. Because the proposed system reads out phase-insensitive intensity reflectivity, time-resolved signals do not produce depth-resolved information.

**Figure 2:**
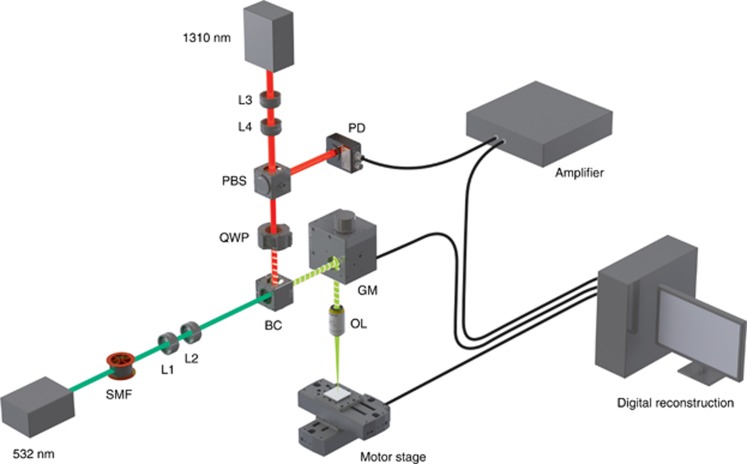
Experimental setup. PARS microscopy with 532-nm excitation and 1310-nm integration beams. BC, beam combiner; GM, galvanometer mirror; L, lens; OL, objective lens; PBS, polarized beam splitter; PD, photodiode; QWP, quarter wave plate; SMF, single mode fiber.

Figure 2 demonstrates the experimental setup of the PARS system. A short coherence length (40 μm) interrogation beam is selected, along with a non-interferometric design to measure intensity oscillations Δ*R*(*t*) and intentionally eliminate sensitivity to phase oscillations. Probe beam reflectivity modulations due to photoacoustic excitation are captured using a photodiode connected to an RF amplifier. To ensure that only initial pressures are recorded, the first 50 samples of data (~250 ns) are captured. The beams can be scanned using 2D galvanometer mirrors to provide real-time imaging, or they can be held stationary while the sample is scanned using a two-axis mechanical motor stage system for larger scan areas.

Briefly, a 532-nm nanosecond-pulsed fiber laser beam was coupled to a single mode optical fiber. A 1310-nm continuous diode laser with a 40 μm coherence length was used to interrogate the reflected light from the sample with a spot that was co-focused with the excitation beam. The collimated interrogation beam passed through a polarized beam splitter (VBA05-1550, Newton, New Jersey, USA) to direct vertically polarized light through a λ/4 zero-order wave plate (Thorlabs Inc., Newton, NJ, USA) into a beam combiner (BC). Then, it was scanned across the samples via a 2D galvanometer scanning mirror system (GVS012/M, Thorlabs, Inc.) along with the excitation beam. The scanning mirrors were driven by a two-channel function generator (AFG3022B, Tektronix Inc., Beaverton, OR, USA). The scanning light was then focused tightly using a 0.4-numerical-aperture objective lens (M Plan Apo NIR 20X, Mitutoyo, Kawasaki-shi, Japan). The light reflected back through the wave plate was converted from circular to horizontal polarization and then reflected at the polarizing beam-splitter interface, directing the maximum possible intensity of reflected light to a 150-MHz bandwidth InGaAs photodiode (PDA10CF, Thorlabs, Inc.). The output of the photodiode was amplified using an RF amplifier (5900PR, Olympus, Center Valley, PA, USA) with a band pass filter (1–50 MHz) and 26 dB gain and then digitized using a four-channel, 12-bit PCI digitizer (CSE1242, Gage Applied, Lockport, IL, USA) at a sampling rate of 200 million samples per second. A two-axis mechanical scanning system was used for imaging larger fields-of-view. Two linear stages (Micos PLS-85, PI, Auburn, MA, USA), each driven by a bipolar microstep driver (Anaheim Automation MBC2508, Anaheim, CA, USA), provided lateral sample movement down to a 1.25 μm step size, while maintaining scanning mirrors in a fixed position. The scanning region size was limited by the onboard capture card memory, though multiple captures or data streaming could be implemented to extend the imaging range to the full reach of the motor stages.

Images which were captured using the galvanometer scanning mirror system used fixed fast and slow scanning rates of 65 and 0.25 Hz, respectively. Large field of view images were formed using 2-axis mechanical scanning and were performed with a 2.5 μm step size at a 2.5 KHz acquisition rate. For example, to produce a 4 × 4 mm scan, roughly 17 min were required. The pulse repetition rate of the excitation laser was fixed at 40 KHz for all of the images shown in the manuscript.

## Results and discussion

**Figure 3:**
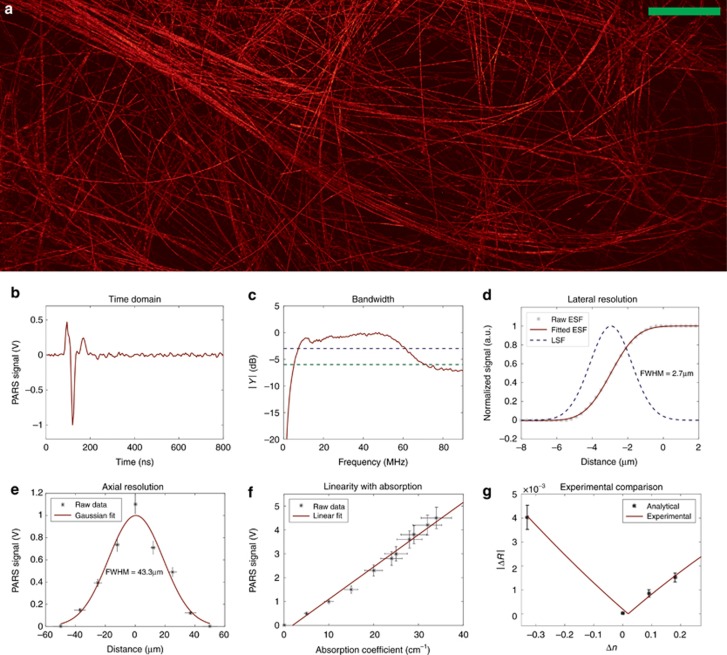
Phantom studies. (**a**) Carbon-fiber network image using mechanical scanning (**b**) Time domain PARS signal due to a single carbon fiber. (**c**) The −3 dB (blue line) and −6 dB (green line) frequency response bandwidth of PARS system by imaging a carbon fiber. (**d**) Line spread function (LSF) and edge spread function (ESF) are presented. The ESF was extracted directly from the captured voltage signal data of a single carbon fiber. From this, the LSF was calculated. The full-width-half-maximum (FWHM) lateral resolution was estimated as 2.7±0.5 μm (*R*^2^=0.999). (**e**) The signal strength as a function of depth measured as FWHM of 43.3±5 μm (*R*^2^=0.894). (**f**) Measured photoacoustic signals from various red dye concentrations producing different absorption coefficients (*R*^2^=0.988). (**g**) Probe beam intensity modulation signals as a function of refractive index contrast. An absorbing bottom layer with a constant refractive index was positioned below several top layers with various refractive indices. The original data of PARS signal versus Δ*n*=(*n*_1_−*n*_2_) is shown in the Table 1 of the Supplementary Information (*R*^2^=0.995). Scale bar: 500 μm.

Figure 3a demonstrates PARS imaging of ~7 μm carbon fiber networks at ~1 mm depth in water. This depth is significantly greater than the 40 μm coherence length of the probe beam, such that any local optical interferometry of reflected light between the target and surface signals was rejected. This large field of view image was captured using ~1 nJ excitation pulse energy and ~4 mW interrogation power on the sample using the mechanical scanning method explained in the previous section. The signal-to-noise ratio (SNR), defined as the average of the maximum amplitude projection pixels in a region of interest over the standard deviation of the noise, was quantified as 60±3 dB.

A representative PARS signal as a function of time is shown in Figure 3b. This was acquired by imaging a single carbon fiber at a depth of approximately 0.25 mm in a 20% intralipid scattering medium (reduced scattering coefficient of ~10 cm^−1^ at 532 nm wavelengths, similar to previous work^43^). The photoacoustic signals were recorded coincident with the laser excitation pulse. Only a 75 ns time delay between PA signals and excitation laser pulses was observed, which is consistent with the group delay of the analog filters of the RF amplifier. When a carbon fiber target was positioned at different depths below a water surface, no additional time-of-flight was measured. This demonstrated that signals were associated with initial pressures rather than propagation-delayed surface oscillations. Such signals were only present when an absorbing target was imaged, eliminating concerns of pump-beam contamination in the photodiode signal. More information about time-of-flight measurements is given in Supplementary Information, Section 2. In addition, to demonstrate that PARS image contrast was due to transient modulations associated with excitation pulse absorption and not inherent scattering, a set of experiments were performed as shown in the Supplementary Information, Section 3.

Figure 3c demonstrates the power spectral density of PARS signals from a single 7 μm carbon fiber placed at 0.25 mm depth in a 20% intralipid scattering medium. The −3 dB (blue line) and −6 dB (green line) bandwidth are measured as 54 and 65 MHz, respectively. These measurements were acquired without an amplifier and with only a 25 KHz analog high-pass filter and a 1 MHz digital high-pass filter. Representative time domain signals are shown in Supplementary Figure 1b. Fourteen hundred A-scan signals from the carbon fiber were averaged for the bandwidth calculation. These time domain signals were windowed with a 300 ns length hamming function around the signal and then zero padded to 200 samples, prior to conversion to frequency domain.

The resolution of the PARS system was characterized by imaging a single carbon fiber (~7 μm diameter). The lateral resolution of the system was measured as approximately 2.7±0.5 μm, as shown in Figure 3d. The edge spread function presented is the raw data collected from the carbon fiber phantom. This was fit to an error function using the MATLAB curve fitting toolbox (normalized mean square error of 2.11 × 10^-5^) from which the line spread function (LSF) was calculated as its derivative.

The same single carbon fiber at different axial depths was imaged to characterize the axial resolution. The signal strength as a function of depth demonstrated a full-width-half-maximum (FWHM) of approximately 43 μm, as shown in Figure 3e. This was consistent with the depth-of-focus of the excitation beam, calculated as 40 μm. Experimental details in the Supplementary Information (Section 2, time-of-flight) illustrate that the current system detected signals from all depths within the interrogation beam depth-of-focus to be coincident in time with the excitation pulse. Thus, time-resolved signals did not provide depth discrimination, and axial resolution was determined by the optical depth-of-focus. Similar to OR-PAM, penetration depth was limited by the ability to focus in the quasi-ballistic range of turbid media to within a transport mean-free path.

To demonstrate that the PARS signal strength was proportional to the optical absorption coefficient, detected intensity modulations as a function of target absorption were measured, as shown in Figure 3f. In this experiment, 200-μm tubes were filled with serial dilutions of an India/ink/water solution. The optical absorption coefficients of the dye mixtures were independently measured with a spectrometer (USB4000, Ocean Optics, Dunedin, FL, USA). In this experiment, PARS signals were acquired using galvanometer scanning mirrors (details are given in a previous section) and averaged over a 0.1 × 0.1 mm region.

To verify that PARS signals were proportional to refractive index contrast, phantom experiments were performed with a variety of simple interfaces. A phantom was made of India/ink-dyed (~10% v/v, *μ*_*a*_=400 cm^−1^) gelatin (*n*~1.33) as the absorbing bottom layer (*n*_1_). For the top layers (*n*_2_), different refractive indices, including air (*n*_2_=1.0), water (*n*_2_=1.33), vegetable oil (Unico Inc., *n*_2_=1.4), and index-matching oil (Cargille Laboratories, Type A, #1248, *n*_2_=1.51), were used. The PARS imaging system using galvanometer scanning mirrors, shown in Figure 2, was used to measure the reflectivity modulation Δ*R*. First, the static backscattered light without the excitation beam was measured over a 0.5 × 0.5 mm region. Then, the same area was scanned using an excitation beam to generate PARS signals. A focal fluence of 500 mJ cm^−2^ was used to produce an estimated index modulation of *δn*=0.038. The final values shown in Figure 3g were calculated by subtracting the static backscattered light from the mean of the maximum PARS signals, as shown Equation 2. Experimental data demonstrated good agreement with the predicted behavior of the model, with *R*^2^=0.995.

To ensure that the signals were not due to ablative, cavitation, or vaporization mechanisms, the focal fluence was maintained below the ablative threshold levels (<1 J cm^-2^), and it was typical of previous OR-PAM systems^42^. With carbon fiber phantoms, only approximately 1 nJ pulses were focused to an ~3 μm spot size, leading to a focal fluence of 14 mJ cm^−2^, which was significantly below the ablation threshold^44^. Although we performed phantom experiments to demonstrate that the source of PARS signal follows model predictions from Equation 1, transient bubble creation could occur for high focal fluences and further contribute to probe beam scattering modulation. Nevertheless, such micro- or nanobubbles were not observed in experiments (Supplementary Information, Section 4), even for focal fluences as high as 500 mJ cm^−2^. Such fluence levels are commonly used in other photoacoustic microscopy systems^42^. Moreover, all of the images shown in this manuscript had limited surface fluences of 20 mJ cm^−^^2^, consistent with the ANSI limit (Supplementary Information, Section 1). A camera system was installed to simultaneously provide reflectance images, and it revealed no evidence of bubble formation (Supplementary Information and Figure 3).

**Figure 4:**
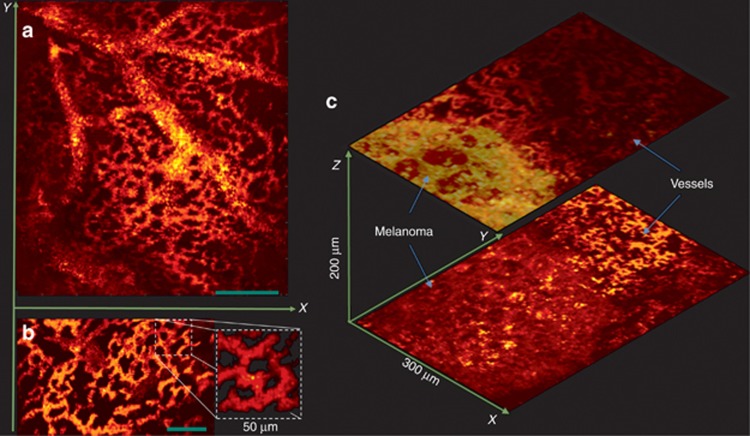
*In vivo* images of the CAM from a chicken embryo. (**a**) En-face C-scan PARS images (**b**) A snapshot of real-time imaging of capillaries at 30 FPS. (Media 1) (**c**) PARS images of a melanoma tumor and surrounding vasculature. Scale bar: 100 μm.

Figure 4a demonstrates *in vivo* images of the chorioallantoic membrane (CAM) of chicken embryos 14 days post incubation at 38 °C. This imaging session was performed using galvanometer scanning mirrors (details are outlined in the Materials and Methods section). In this model, larger blood vessels are located deeper than capillaries. Confocal microscopy images of fluorescently labeled microvasculature in the CAM were acquired in the same chicken embryo (Supplementary Information, Section 5), and they were comparable with label-free PARS images.

Figure 4b demonstrates a snapshot of real-time imaging of capillary beds at 30 frames per second (FPS; Media 1). To achieve real-time imaging, the field of view was restricted to ~50 μm, and the laser pulse repetition rate was set to 600 KHz with 15 Hz and 1.2 KHz slow and fast axis galvanometer mirror scanning rates, respectively. Real-time implementation of the scanning mirror captures were performed using the same hardware. The data acquisition card was operated in a data streaming mode (eXpert FPGA DSP, Gage Applied), which was interpreted in real-time by software developed in house using the Gage Applied C/C++ SDK. To maintain higher frame rates, a more basic scatter point interpolation was used, which resulted in lower resolution over single captures. Translational motion observed in the video was ascribed to subtle embryo motion captured over the 2 s observational window. Intensity fluctuations were attributed to red-blood cell number density variability in the small vessels observed. Further development work, including faster data steaming and multi-threaded implementation of analysis, would generate improvements in performance; however, this technique is ultimately limited by the maximum tilt speed of the fast scanning mirror axis.

Melanoma tumor imaging was performed to demonstrate the capabilities of PARS for imaging melanin. Shell-less chicken embryos with tumors in the CAM were prepared as previously described^45^. Fertilized chicken eggs were placed in a humidified 38 °C rocking incubator for 3 days, cracked into plastic weigh boats, and placed in a humidified 38 °C incubator for five days. B16F10 cells were injected intravenously (~100 000 cells) and allowed to form multiple metastatic sites, and then they were imaged seven days later. PARS images of a melanoma tumor are shown in Figure 4c. Melanoma xenograft PARS images were simultaneously imaged with a co-registered brightfield camera system to confirm location. The capillary beds surrounding the tumor were easily distinguishable from the melanin signature of the melanoma tumor using PARS imaging. Scanning at multiple depths clearly revealed heterogeneity between blood vessels and throughout the melanoma tumor.

**Figure 5:**
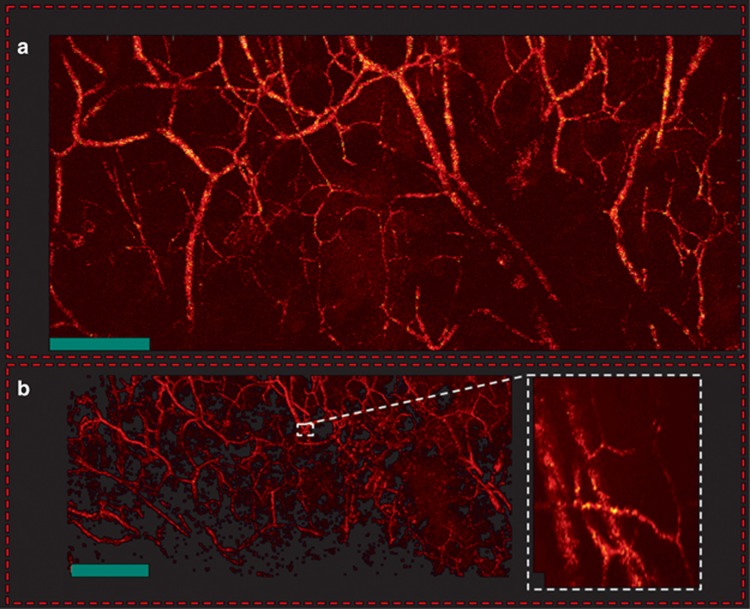
*In vivo* en-face mouse ear images. (**a**) PARS images using two-axis lateral mechanical scanning. (**b**) Larger field of view images using mechanical scanning as well as a zoomed in image of both capillary beds and larger blood vessels using fast scanning mirrors. Scale bar: 500 μm.

Figure 5 demonstrates *in vivo* PARS images of en-face microvasculature in the ear of an 8-week-old nude mouse (NU/NU, Charles River, MA, USA) using a two-axis lateral mechanical scanning stage (as explained in a previous section). In all *in vivo* imaging, a pulse energy of ~40 nJ was used with interrogation power fixed at 4 mW. The SNR of the large vessels (average of maximum amplitude projection pixels in a region of interest over the standard deviation of the noise, measured in a separate region with no vessels) was measured as approximately 40 dB, which was comparable to the ~40 dB SNR reported previously using contact or liquid-coupling second generation OR-PAM with 80 nJ pulse energy^46^. Local intensity variations within vessels, seen in Figures 4 and 5 were attributed to the red-blood cell concentration variability. Such variability may be expected because the lateral resolution was comparable to the size of erythrocytes.

All of the experimental procedures were carried out in conformity with the laboratory animal protocol approved by the University of Alberta Animal Use and Care Committee. Authors were also trained and certified to use mice and rats in the research work. During the imaging sessions, animals were anaesthetized with isoflurane using a breathing anesthesia system (E-Z Anesthesia, Euthanex Corp., Palmer, PA, USA). All of the 2D images shown in this manuscript were formed using a maximum amplitude projection (MAP) from the Hilbert transform of each A-scan as a pixel in a C-scan en-face image. All images shown in this manuscript were produced either by direct plotting of raw data (mechanical scanning of the sample) or from interpolated raw data (mirror scanning of interrogation spot) followed by 3 × 3 median filtering. In the case of galvanometer scanning, a Delaunay triangulation interpolation algorithm was used to render the data acquired on a sinusoidal trajectory onto a Cartesian grid. All image and signal processing steps were performed in the MATLAB environment.

Many future opportunities may be realized by capitalizing on this mechanism of optical imaging of absorption in reflection mode. OR-PAM previously relied on acoustic coupling media and ultrasonic detectors to form high-quality images of optical absorption at superficial depths. PARS microscopy offers an all-optical non-contact approach that no longer requires a coupling medium. PARS uses laser fluence similar to or less than previous OR-PAM systems, and laser exposure on the skin can be less than the maximum permissible exposure limits, as discussed in Section 1 of Supplementary Information. The all-optical nature of the imaging may enable future combinations with other optical modalities such as OCT, fluorescence microscopy, and others. Many of the fascinating developments and applications of OR-PAM, including real-time functional brain imaging, single-cell and super-resolution imaging and visualization of circulating tumor cells, may be achievable in a non-contact implementation with future developments of the PARS technology. The all-optical nature of the scanning also removes the requirements for scanning of single-element focused transducers used for best-quality PAM images. Future work will aim to further extend PARS capabilities, improve resolution, demonstrate functional multi-wavelength imaging, image flow, and further improve real-time capture rates. Future work could also aim to add interferometry to decouple amplitude and phase effects and provide coherence-gated depth resolution. Clinical applications in dermatology, gastroenterology, ophthalmology, and other fields are anticipated.

## Conclusion

Photoacoustic-induced reflectometry was demonstrated in phantoms and *in vivo* using a novel photoacoustic remote sensing microscope. PARS signal contrast is proportional to both the local refractive index step and the optical absorption coefficient. The non-interferometric nature of the system and the low-coherence of the interrogation beam rejects phase modulations from the surface and other locations, such that the PARS signal is attributed to initial pressure waves at subsurface absorber locations. High signal-to-noise ratio *in vivo* images were achievable in real-time. The present work may provide the basis for a large number of biological and clinical applications and add complementary contrast to other optical imaging modalities.

## Author contributions

PH discovered the PARS imaging effect, designed and developed the system, built and carried out imaging experiments and drafted the manuscript. WS helped design and develop the system, performed theoretical analyses, helped elucidate the PARS mechanism, worked on *in vivo* imaging experiments, and helped with software and image processing. KB developed mechanical scanning hardware, software and image processing and helped with experiments. RJP created the tumor-bearing animals used for imaging and obtained fluorescent images of chorioallantoic membrane vasculature. RJZ scientifically directed the project, co-designed the system, co-designed experiments and elucidated and modeled the PARS mechanisms. All authors contributed to the writing of the manuscript. PH and RZ have financial interests in illumiSonics, Inc.
