# Supplementary material for: Non-interferometric photoacoustic remote sensing microscopy
Source: Light Sci Appl. 2017 Jun 2;6(6):e16278–. doi: 10.1038/lsa.2016.278 (PMC6062239; doi:10.1038/lsa.2016.278)
Supplement: Supplementary Information [file lsa2016278x1.docx]

**Non-Interferometric Photoacoustic Remote Sensing Microscopy**

Parsin Hajireza^1,2^, Wei Shi^1^, Kevan Bell^1^, Robert J. Paproski^1^, and Roger J. Zemp^1,2^

1. *Department of Electrical and Computer Engineering, University of Alberta, Edmonton, Alberta, T6G 2V4, Canada*
2. *illumiSonics Inc., 5205-38A Ave. N.W. Edmonton, AB, T6L 2J4*

*parsin@illumisonics.com* and *[rzemp@ualberta.ca](mailto:rzemp@ualberta.ca)*

1. **Laser Safety**

*1.1 Excitation-Beam Laser Safety*

The pulse energy of the excitation beam for *in vivo* PARS imaging has been set as 40 nJ. In our *in vivo* studies, given that the optical focus is ~ 150 μm beneath the tissue surface, with a focal spot of ~3 µm in diameter, the surface spot size is ~ 100 µm in diameter for a 0.4 Numerical Aperture lens and the calculated surface laser fluence is ~0.5 mJ cm-2 for excitation laser, which is below the single pulse limit of 20 mJ cm-2 set by the American National Standards Institute (ANSI) [1]. The spatial peak optical fluence at the focus is ~560 mJ cm-2, which is still less than the damage threshold observed in small animals and comparable to other OR-PAM systems [2]. In our work, light delivery is confined to a localized area and no tissue damage is visible after imaging. In addition, for an average scan-step size *Δx* (average distance between excitation laser shots on the sample during galvanometer scanning) of 3 µm, the average number *N* of adjacent laser pulses overlapping on the skin surface can be obtained by dividing the surface spot size (100 µm) by scan-step size (3 µm): *N* = 100 µm/3 µm ~ 33. For 40 kHz laser PRR (as was used to acquire Figure 4a,c, the exposure time ( *t* = *N*/40k ) is ~0.8 ms, so the Maximum Permissible Exposure (MPE) for a pulse train is *MPE* _Train_ = $1.1 C_{A}t^{0.25}$ = 185 mJ cm-2, where $C_{A}$ is a wavelength-correction factor equal to unity for 400 - 700 nm wavelengths [1]. The average power limit set by ANSI is calculated as *MPE* _Average_ = *MPE* _Train_/*N* which is ~ 6 mJ cm-2. Thus our actual surface fluence of ~ 0.5 mJ cm-2 is about 12 times lower than the ANSI limit.

*1.2 Interrogation beam laser safety considerations*

Optical scanning on a 1 mm x 1 mm or smaller area with a laser power of 4 mW was performed. For CW lasers with wavelengths between 400 to 1400 nm, and exposure times between 100 ns and 10 s, MPE on the skin is limited to a deposited energy density of $MPE=1.1 C_{A}t^{0.25}$, where $C_{A}$ is a wavelength-correction factor equal to 5 for 1050 - 1400 nm wavelengths [1]. When considering a fast-axis galvanometer scanning rate of 60 Hz and a slow-axis scanning rate of 0.25 Hz (half C-scan frames per second) over 1 mm field-of-view (used for Figure 4a,c), the average power limit set by ANSI is calculated as $MPE_{Average} =MPE/t$~ 3 W cm-2, where *t* is 2 seconds for the whole FOV. Thus our actual surface average power of 0.4 W cm-2 is about 8 times lower than the ANSI limit. In addition, laser beam scanning on the skin surface may need to be considered. If the average scanning speed is $v$ = 133 mm s-1 (as is the case for a 60 Hz sine-wave scanning trajectory over 1 mm FOV) and the 1310-nm light-spot has a diameter $d$ of 100 μm and 800 μm (for subsurface focal depths ~ 150 μm and ~ 1 mm) on the skin surface then the exposure time $T_{e}=d/v$ associated with the scanning trajectory sweeping a path-length $d$ are 0.8 ms and 6 ms (assuming only one scan-line in a C-Scan is acquired). When acquiring one C-scan image, any given area will experience on average *N* ~$d/\Delta y$ passes of the probe-spot (*N* is 25 and 200 for focal depths at ~ 150 μm and ~ 1 mm respectively) where $\Delta y$ is the mean y-scanning resolution ~ 4 μm. Thus the exposure time is $T_{\mathrm{EXP}}={NT}_{e}$ and leads to ${MPE}_{A\mathrm{verage}}=MPE/T_{\mathrm{EXP}}=1.1C_{A}T_{\mathrm{EXP}}^{0.25}/T_{\mathrm{EXP}}$ of 103 W cm-2 and 5 W cm-2 for focal depths of ~ 150 μm and ~ 1 mm. In our experiment, the fluence on the skin surface can be calculated ~ 51 W cm-2 and ~ 1 W cm-2 for focal depths at ~ 150 μm and ~ 1 mm respectively, which are ~ 2 times and ~ 5 times lower than the ANSI limit, respectively. Considering that only ~ 1 μs of measurement time is needed for each laser pulse (equivalent to ~ 1.5 mm of depth), the interrogation beam can be modulated to be active only during the measurement phase to reduce the fluence rate by more than 100 times. Additional improvements in signal-to-noise and detection sensitivity could also enable lower incident laser power levels. Future work will pursue such directions.

1. **Time of Flight**


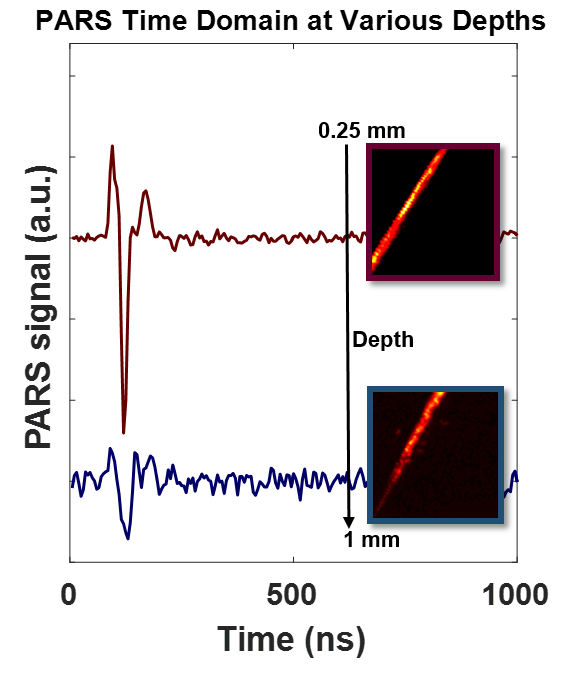


**(b)**

**(a)**

**Figure S1 Time of flight. Figure S1a,** Time domain signal of a single carbon fiber positioned at different depths to show PARS signals occur at time zero independent of the depth of the target. Note that there is a 75 ns time-delay associated with the analog filters. **Figure S1b,** Time domain signal of a single carbon fiber at 0.25 mm depth without any amplifier and only a 25 kHz analog high pass filter and 1 MHz digital high-pass filter, unlike **figure S1a** which used an amplifier with 26 dB gain and a 1 - 50 MHz bandpass filter. **Figure S1b** exhibits only a ~ 10 ns time delay and shows broader frequency response as plotted in figure 3c of the main text.

Figure S1a shows that PARS signals occurred coincident with the excitation pulse, referred to as time zero, even when the targets were placed at varying depths below the surface. 75 ns time delay between PA signals and excitation laser pulses was observed which is due to the group delay of the analog filters of the RF amplifier, verified independently with a function generator. In this experiment a single carbon fiber has been placed at 0.25 mm and 1 mm depth by using 20% intralipid scattering medium (reduced scattering coefficient of ~ 10 cm-1 at 532nm wavelengths, similar to previous work [3]). The effective delay is significantly less when no amplifier or filter is used as shown in figure S1b.

1. **Absorption versus Scattering Contrast**

In order to demonstrate that PARS image contrast is due to transient modulations associated with excitation-pulse absorption and not inherent scattering, we imaged a carbon fiber network embedded at ~ 1 mm depth in a 20% intralipid scattering medium having a reduced scattering coefficient of ~ 10 cm-1 [4]. Supplementary figure S2a is formed using the PARS imaging system demonstrated in figure 2 of the manuscript. It shows when the excitation beam (5 nJ pulse energy) is present, carbon fiber networks are visualized with high absorption contrast. Figure S2b is formed using the same system while the excitation beam was blocked. It represents modulations of native backscattered light and shows no absorption contrast. The interrogation beam power was fixed at 5 mW and a 20 kHz - 50 MHz in-line filter was used in both cases. Figure S2c and 2d are formed using identical settings but without any filter. Figure S2d represents laser speckle detected in the intralipid solution, unlike Figure S2b which represents the changes in this speckle pattern that pass through the filter and may in part be due to the detection beam scanning across the target. The presence of the filter improves SNR (SNR of 46 dB in figure S2a versus 36 dB in figure S2c) by capturing only modulated signals due to absorption contrast. The units of the colorbars shown are peak-to-peak volts of measured PARS signal.


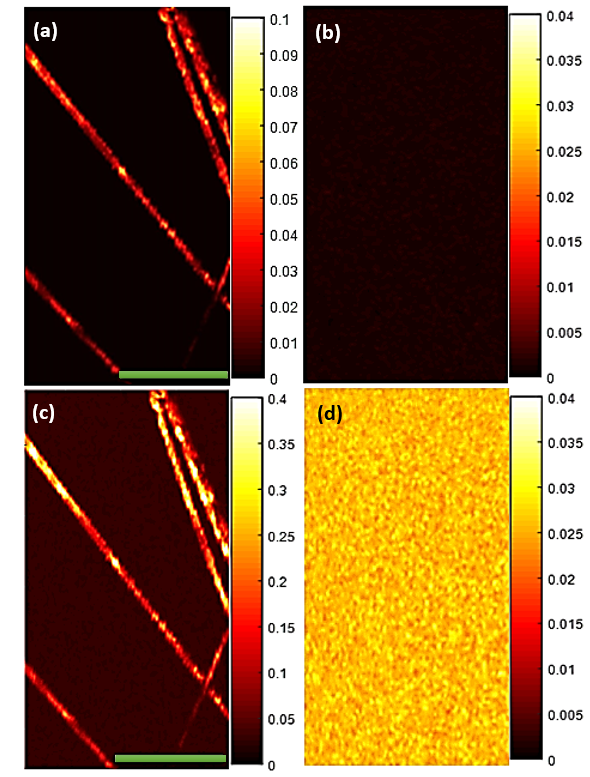


**Figure S2 Absorption contrast validation. Figure S2a.** PARS image of carbon fiber networks **Figure S2b.** Image due to native backscatter fluctuations when the excitation beam is blocked. **Figure S2c.** A new scan in the same location as **figure S2a** when no filter is used. **Figure S2d.** A repeat of the scan shown in f**igure S2b** when no filter is used.

1. **Investigating the Possibility of Bubble Generation**

A camera system is used to image targets simultaneously with PARS system. As shown in media 2 and figure S3b no bubbles were generated while the figure S3a (original PARS image) was formed. This is evidence that PARS imaging can be performed at photoacoustic excitation energies which do not create any visible cavitation or bubble generation. A filter has been placed in front of the CCD camera to reject 532 nm used to generate photoacoustic signals. However the scanning excitation light still can pass through the filter. The intensity changes seen in the video (media 2) are due the scanning excitation light.


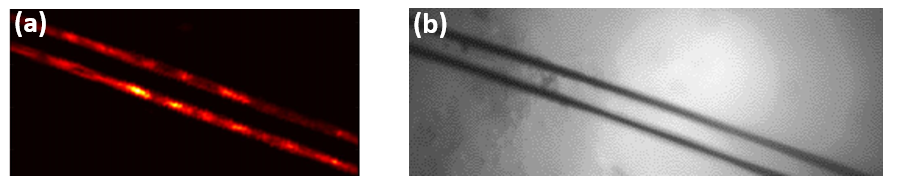


**Figure S3 Carbon fiber images.** **Figure S3a,** PARS image **Figure S3b,** A snapshot from the co-aligned camera taken simultaneously with PARS image shown in **figure S3a** (media 2).

In order to further evaluate whether microbubbles or nanobubbles might be a source of probe beam modulation over timeframes shorter than permitted by the camera system, we examined the linearity of the PARS signal with excitation fluence levels as shown in supplementary figure S4. The PARS signal amplitude was observed to be linear, even with fluences as low as 7mJ cm-2, where initial pressures are predicted to be only 1.6 MPa for blood. If bubbles were generated, there should be an expected cavitation threshold which should be reflected in a nonlinear or step behavior in the backscattered light as excitation fluence is increased. It would also give signal deviation from the model predictions shown in figure 3g of the main manuscript. The observed linear behavior is evidence that bubbles are not significantly contributing to PARS signals. Even though initial pressures can be very large, the frequency content of these pressure waves is very high, limiting effective mechanical index (MI), a predictive measure of the likelihood of cavitation [5].

**Figure S4 linear response.** The photoacoustic signal as a function of focal fluence. (Fitting R^2^ of 0.995)

1. **Comparison with Fluorescence Confocal Microscopy**

Figure S5 shows confocal microscopy images of the CAM membrane of a chicken embryo. Some images have extended focus to see vessels at multiple depths. The chicken embryo was injected intravenously with Rhodamine-conjugated lectin to stain the blood vessels. The confocal microscope used was a Zeiss AxioExaminer Z1 microscope with 6 diode lasers, a Hamamatsu ImagEM backthinned EM-CCD camera, environmental chamber and fully motorized XYZ translation built by Quorum Technologies. 496 nm laser/20x objective lens was used for image acquisition. The confocal fluorescence microscopy images of the CAM microvasculature are structurally similar to the PARS images. However, unlike PARS, in fluorescence confocal microscopy, the chicken embryo had to be held stationary during the imaging section as the heartbeat of animal can cause major image defects. In addition, unlike the confocal microscopy approach, the PARS images are label free.


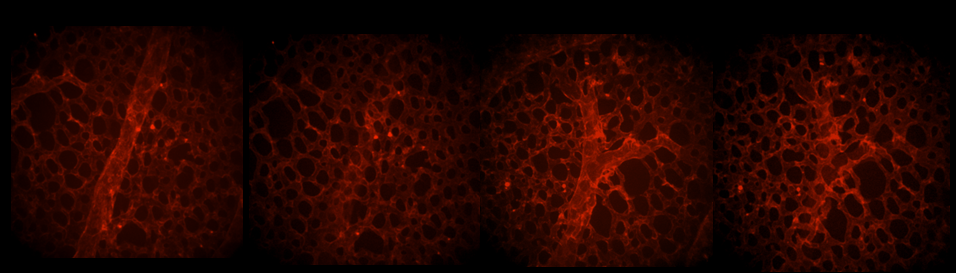


**Figure S5 Confocal fluorescence microscopy images.** Vasculature in the CAM of a chicken embryo

1. **Appendix**

| n_2_-n_1_ | |ΔR| | Error |
| --- | --- | --- |
| -0.33 | 0.004029 | 0.000504 |
| 0.00 | 2.88×10^-5^ | 3.00×10^-5^ |
| 0.09 | 0.000853 | 0.000155 |
| 0.18 | 0.001518 | 0.000186 |

**Table 1** Experimental data from figure 3g of the main manuscript

**References**

1. Laser Institute of America. ANSI Z136.1-2007 American National Standard for Safe Use of Lasers. ANSI, 2007.
2. Maslov K, Zhang FH, Hu S, Wang LV. Optical-resolution photoacoustic microscopy for *in vivo* imaging of single capillaries. *Opt Lett* 2008; **33**: 929-931.
3. Shi W, Kerr S, Utkin I, Ranasinghesagara J, Pan L *et al*. Optical resolution photoacoustic microscopy using novel high-repetition-rate passively Q-switched microchip and fiber lasers. *J Biomed Opt* 2010 ;**15**: 056017.
4. Wang LV, Wu H. *Biomedical Optics: Principles and Imaging*. Wiley: Hoboken, 2007.
5. Apfel RE, Holland CK. Gauging the likelihood of cavitation from short-pulse, low-duty cycle diagnostic ultrasound. *Ultrasound Med Biol*1991; **17**:179-185.
